# Supplementary material for: PIP2-mediated oligomerization of the endosomal sodium/proton exchanger NHE9
Source: Nat Commun. 2025 Mar 28;16:3055. doi: 10.1038/s41467-025-58247-x (PMC11953442; doi:10.1038/s41467-025-58247-x)
Supplement: Supplementary file 1 — Supplementary Information [file 41467_2025_58247_MOESM1_ESM.pdf]

Supplementary information

PIP_2-mediated oligomerization of the endosomal sodium/proton exchanger NHE9

Surabhi Kokane[1*], Ashutosh Gulati[1*], Pascal F. Meier[1*], Rei Matsuoka[1*], Tanadet Pipatpolkai[2], Giuseppe Albano[3], Tin Manh Ho[3], Lucie Delemotte[2], Daniel Fuster[3#], David Drew[1#]

[1]Department of Biochemistry and Biophysics, Science for Life laboratory, Stockholm University, Stockholm, Sweden. [2]Department of Applied Physics, Science for Life Laboratory, KTH Royal Institute of Technology, Stockholm, Sweden. [3]Department of Nephrology and Hypertension, Inselspital, Bern University Hospital, University of Bern, Bern, Switzerland

*These authors contributed equally

[#]Correspondence: daniel.fuster@unibe.ch; ddrew@dbb.su.se

This file contains Supplementary Figures 1-11 and Supplementary Table 1

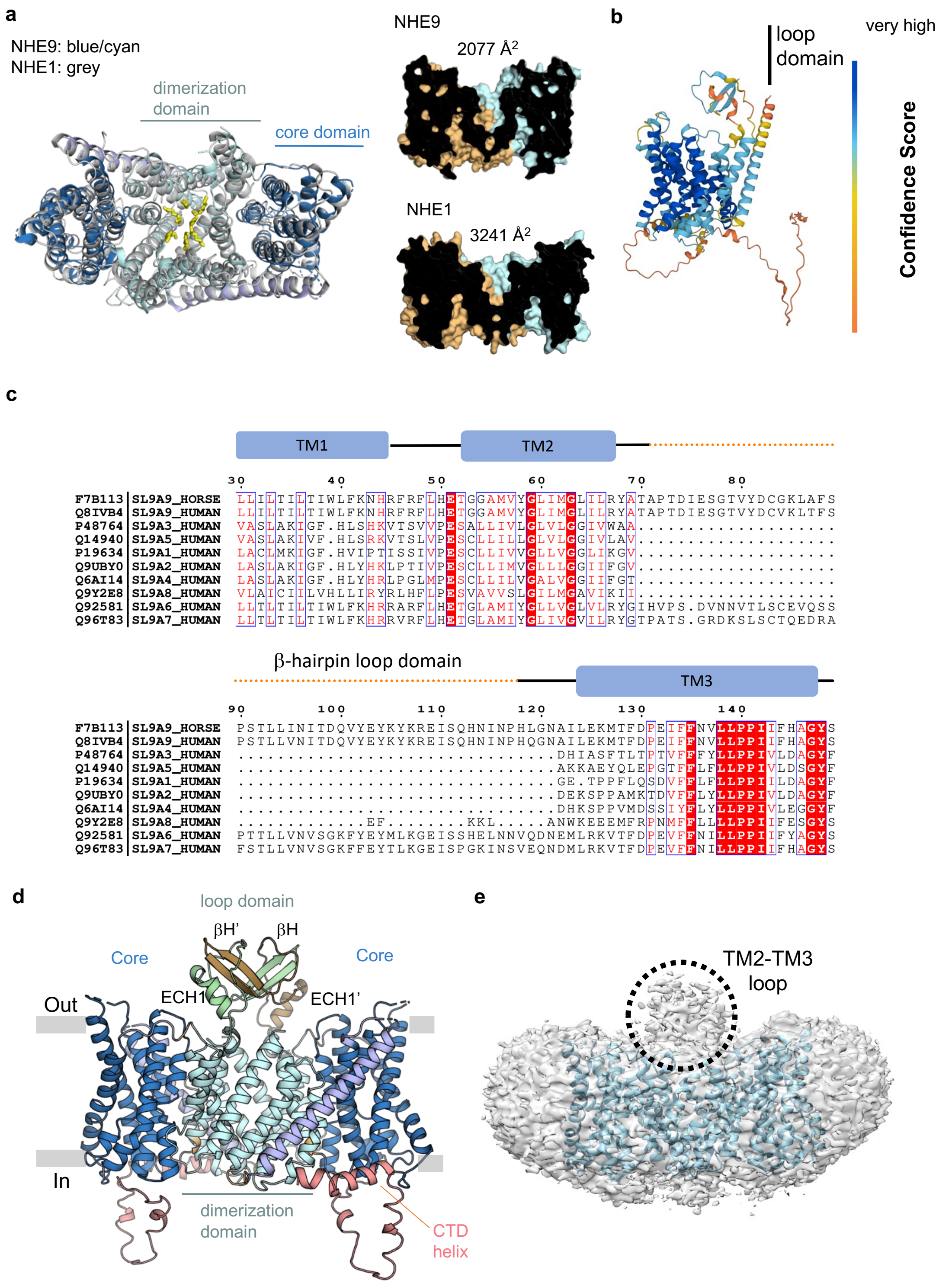

Supplementary Fig. 1. AlphaFold2 model for endosomal NHE9 includes β-hairpin TM2-TM3 loop domain. **a** *left:* Structural superimposition of *E. caballus* NHE9* with inward-facing human NHE1 (C_α RMSD = 2.91 Å) showing the overall structural conservation. Inward-facing NHE9 ΔCTD (PDB: 6Z3Z) with dimerization domain (cyan), core domain (blue) and TM7 linking helix (light-purple) all shown as cartoon. Inward-facing NHE1 (PDB: 7DSW) (grey, cartoon) with lipid tails (yellow, sticks) at

the dimerization interface *right:* Side-view showing the surface representation of *E. caballus* NHE9* (above) and inward-facing human NHE1 (below) homodimer coloured with the protomers coloured in sand and cyan. The buried surface area (Å^2) of the dimerization interface was calculated by the PDBePISA server. **b** AF2 model of the human NHE9 monomer predicts an additional TM2-TM3 loop domain containing a β-hairpin with reasonable confidence. **c** The human NHE1-9 sequence alignment for TM1 to TM3. Residues with over 90% sequence identity are shown in red. The β-hairpin TM2-TM3 loop is only present in organellar NHE6, NHE7 and NHE9 proteins. **d** AF2 model of the *E. caballus* NHE9 dimer with an TM2-TM3 loop domain containing an β-hairpin (βH1, βH1') and extracellular helix (ECH1, ECH1') from each of the two protomers coloured in brown and green, respectively. The core domain (dark-blue), dimerization domain (light-blue) and CTD domain (salmon) is further highlighted. **e** NHE9* structure and maps (PDB: 6Z3Z, EMD: EMD-11067) at low contour levels to highlight the loop domain map features that could not be modelled (black-dotted circle).

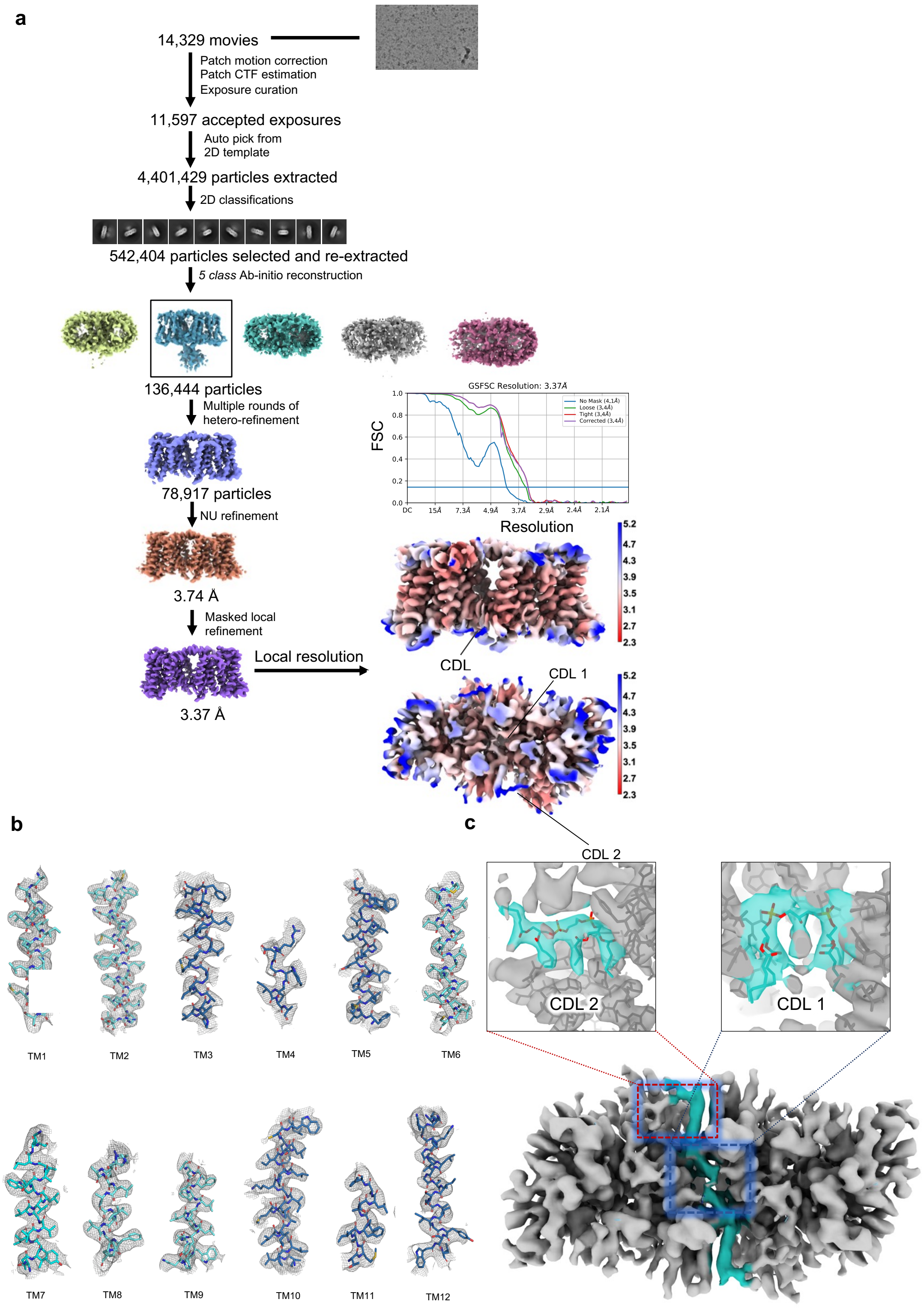

Supplementary Fig. 2. The data-processing workflow of *EcNhaA-mut2* at pH 7.5. **a.** The dataset contained 14,329 movies that were corrected by patch motion correction and patch CTF estimation. After reference-based auto-picking, 4,401,429 particles were picked. Several rounds of 2D classifications yielded in good 2D classes of 542,404 particles. Heterogeneous and non-uniform refinement resulted in an electron density

map of 3.74 Å. Masked local refinement resulted in the final resolution of 3.37Å at the gold-standard FSC (0.143), with a local resolution range of 2.4–3.6 Å. **b.** *left*: cryo-EM density map and model are shown for all the transmembrane segments for NhaA with the dimer domain (cyan), transport domain (sky-blue), all shown as sticks. *right*: cryo-EM density map and cardiolipin lipids (yellow-orange).

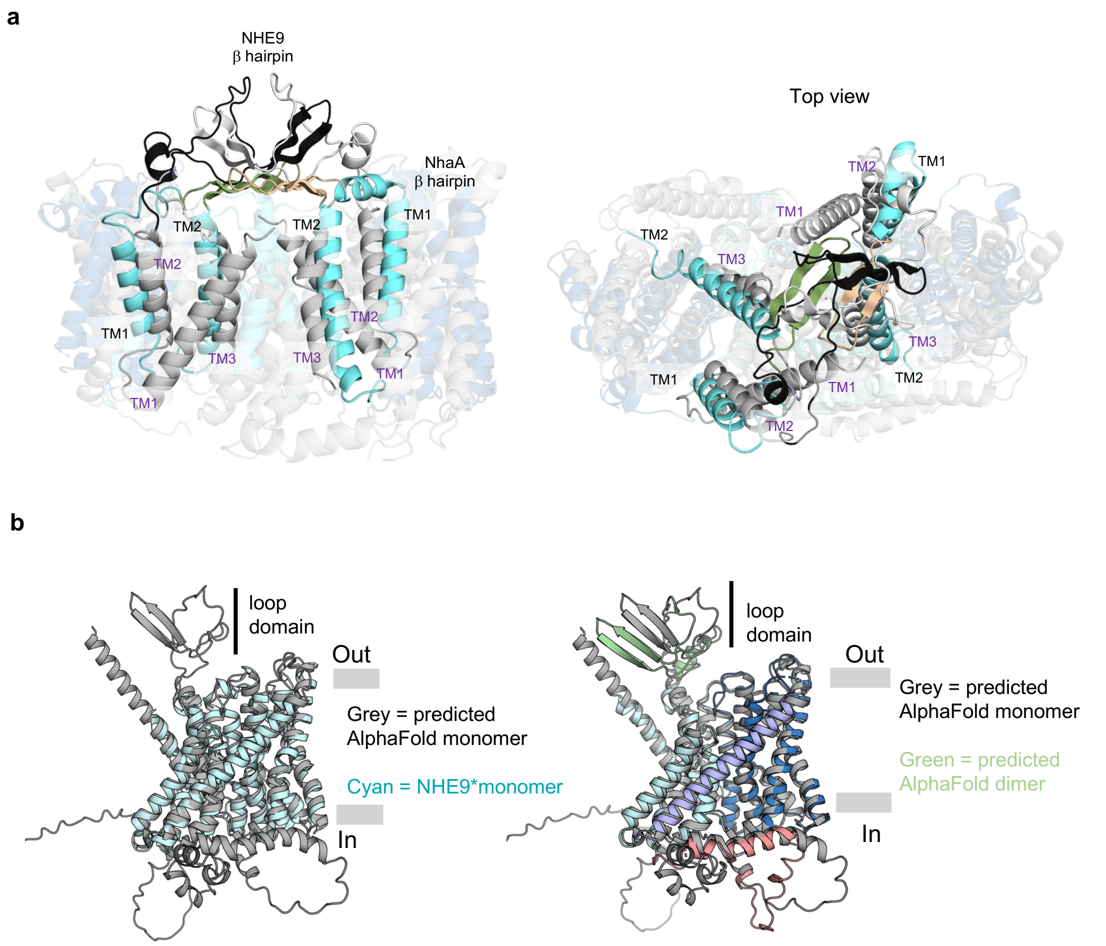

Supplementary Fig. 3. β-hairpin containing NhaA and NHE9 proteins. a *left*: Superimposition of *Ec*NhaA dimer structure (coloured) and NHE9*CC dimer structure (grey) highlighting the topologically equivalent TMs connected to β-hairpin in both the structures. The β-hairpins of *Ec*NhaA (olive and wheat) are oriented parallel to membranes compared to NHE9*CC (black and grey) in a vertically upright position. TMs of *Ec*NhaA (cyan) are marked in black whereas for NHE9*CC (grey) in purple. *right*: Topview of superimposed *Ec*NhaA dimer structure (coloured) and NHE9*CC dimer structure (grey). **b** *left*: Superimposition of the AlphaFold2 *E. caballus* NHE9 monomer and *E.caballus* NHE9 cryo-EM structure (PDB: 6Z3Z). *right*: Superimposition of the AlphaFold2 predicted *E. caballus* NHE9 monomer and dimer models.

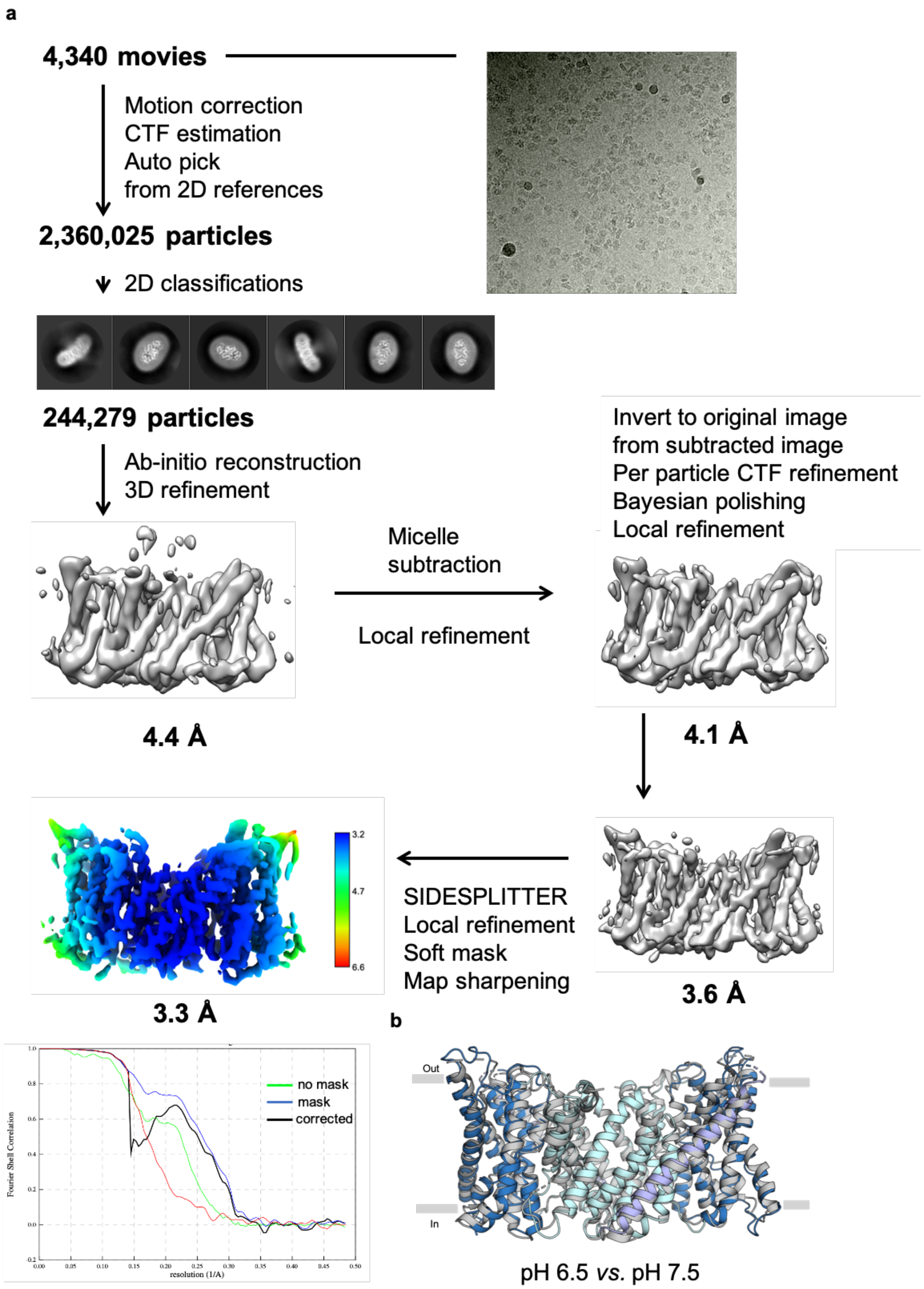

Supplementary Fig. 4. Data-processing workflow of *E. caballus* NHE9*at pH 6.5. **a.** The dataset contained 4,340 movies that were corrected by MotionCor2 and CTFFind. After reference-based auto-picking, 2,360,025 particles were picked. Several rounds of 2D classifications yielded in good 2D classes of 244,279 particles. 3D

classification and 3D refinement of model resulted in an electron density map of 4.4 Å. The electron density map was subjected to micelle subtraction, local refinement, per particle CTF and Bayesian polishing. A final resolution of 3.31Å was achieved at gold-standard FSC (0.143), with a local resolution range of 3.2–6.6 Å. **b**. Superimposed structures of NHE9* at pH 7.5 (grey; PDB ID: 6Z3Z) and NHE9* at pH 6.5 (both as cartoon), with dimerization domain (cyan), core domain (blue), loop domains (pale-green, sand), and TM7 linking helix (light-purple); the β-hairpin TM2-TM3 loop domain in the later modelled NHE9* structure at pH 6.5 is not shown here as it was not observed after masked refinement.

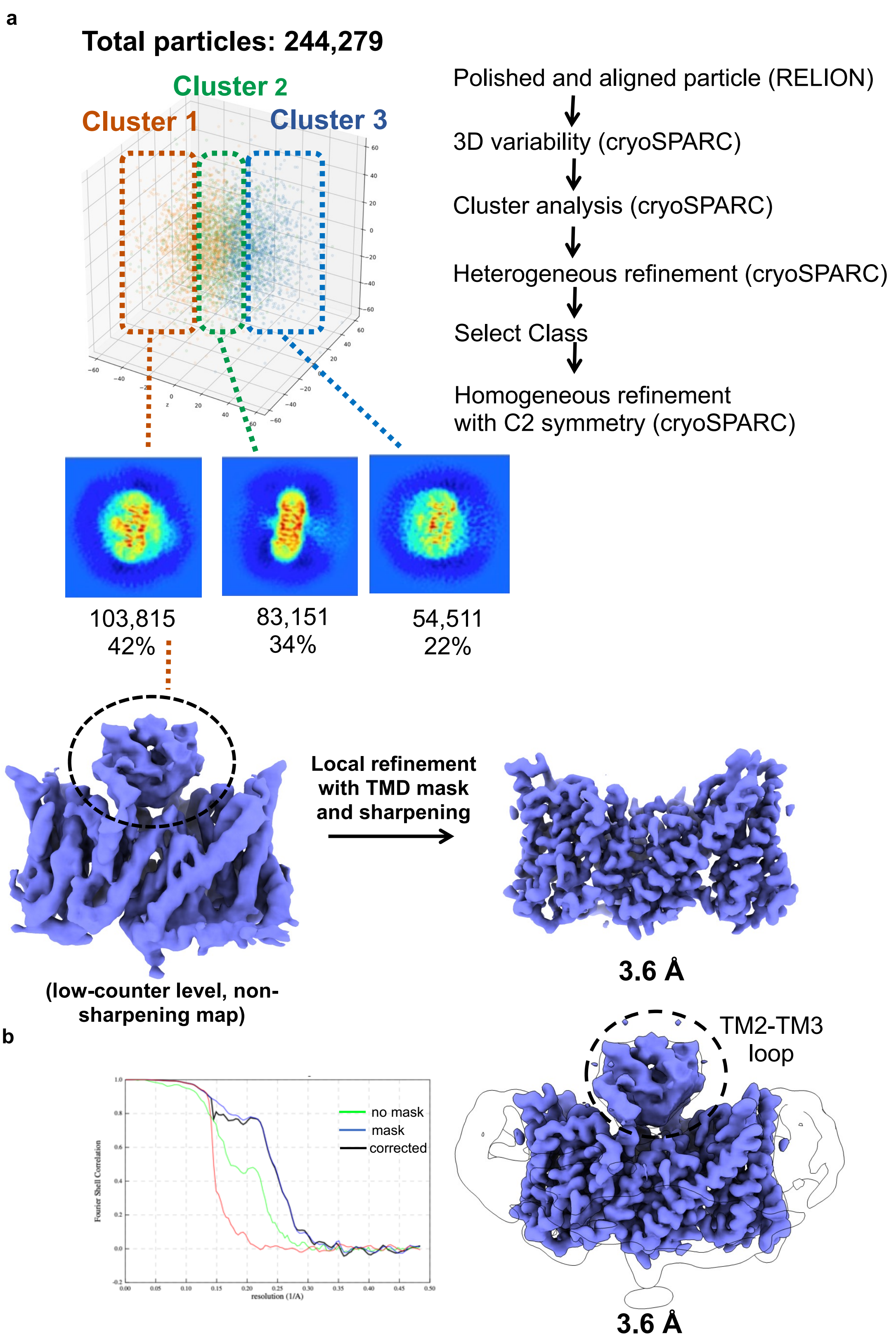

Supplementary Fig. 5. 3D Variability Analysisof NHE9* improved the overall resolution and the density for the TM2-TM3 β-hairpin loop domains. a. All particles of the final 3D reconstruction were subjected to 3D variability analysis followed by cluster analysis. Cluster 1 including 42% of particles were subsequently

subjected to heterogeneous refinement followed by homogeneous refinement with C2 symmetry applied, revealing map features for the TM2-TM3 β-hairpin loop domains. These map features could not be retained after masked refinement. **b.** Composite cryo-EM map combining the map features before and after masked refinement with a final resolution of 3.6 Å at gold-standard FSC (0.143). Improved map density for the TM2-TM3 loop domain above the dimerization domain (black dashed line) was observed.

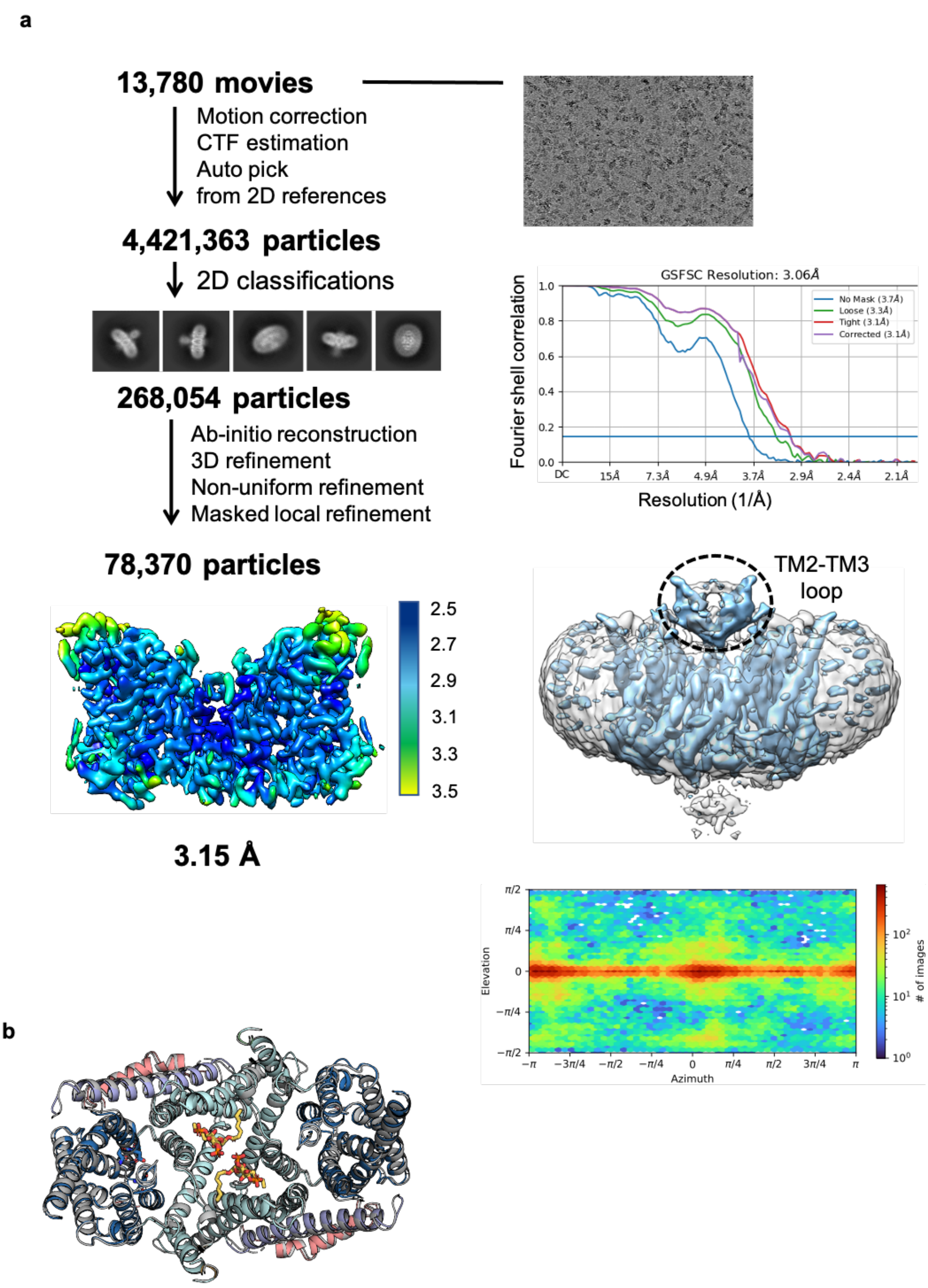

Supplementary Fig. 6. Cryo-EM workflow and NHE9*CC structure. a The dataset contained 13,780 movies that were corrected by patch motion correction and patch CTF estimation. After reference-based auto-picking, 4,421,363 particles were picked. Several rounds of 2D classifications yielded in good 2D classes of 133,145 particles. 3D classification, 3D refinement of the electron density map followed local refinement

resulted in a final resolution of 3.15 Å at gold-standard FSC (0.143). **b.** Superimposed cryo-EM strucure of NHE9*loop (grey) and a subsequent variant NHE9*CC with core domain (cyan), transporter domain (blue), linker helix (light blue), CTD helix (salmon) and the PI(3,5)P_2 lipids as yellow sticks, with protein shown as cartoon.

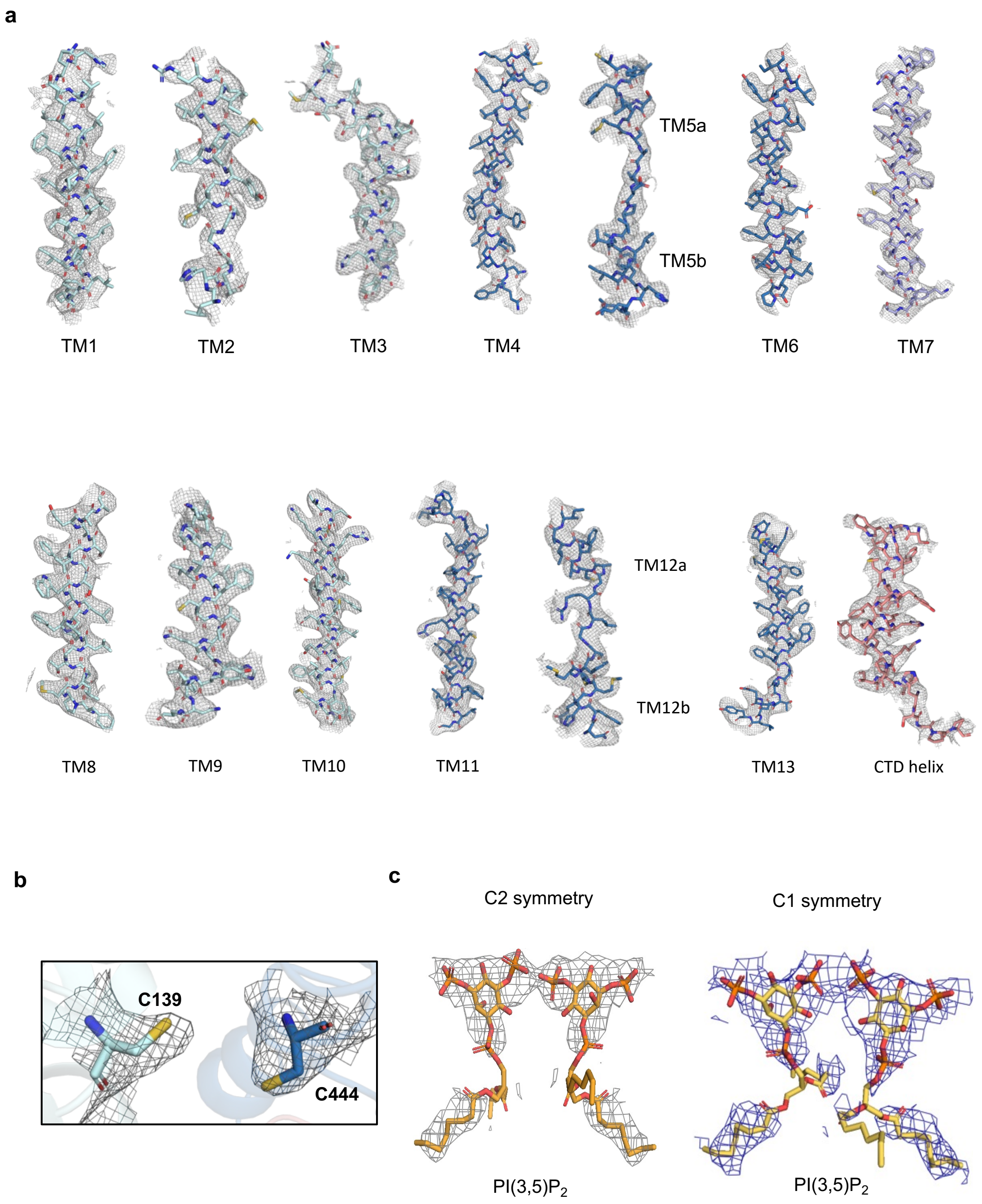

Supplementary Fig. 7. Cryo-EM desity of NHE9*CC. a. Cryo-EM desity map and model are shown for all the transmembrane segments for NHE9* in dimer domain (palecyan), transport domain (blue), and linker helix (lightpurple), helix of the CTD (salmon), all protein shown as sticks colored as in Fig. 1A. **b.** Electron density map (grey mesh) of the two mutated cysteine residues L139C and I444C does not show a disulphide bond. **c.** Cryo-EM map density around the lipid PI(3,5)P_2 (yellow sticks), with (grey mesh)and without C2 symmetry (blue mesh) applied during processing.

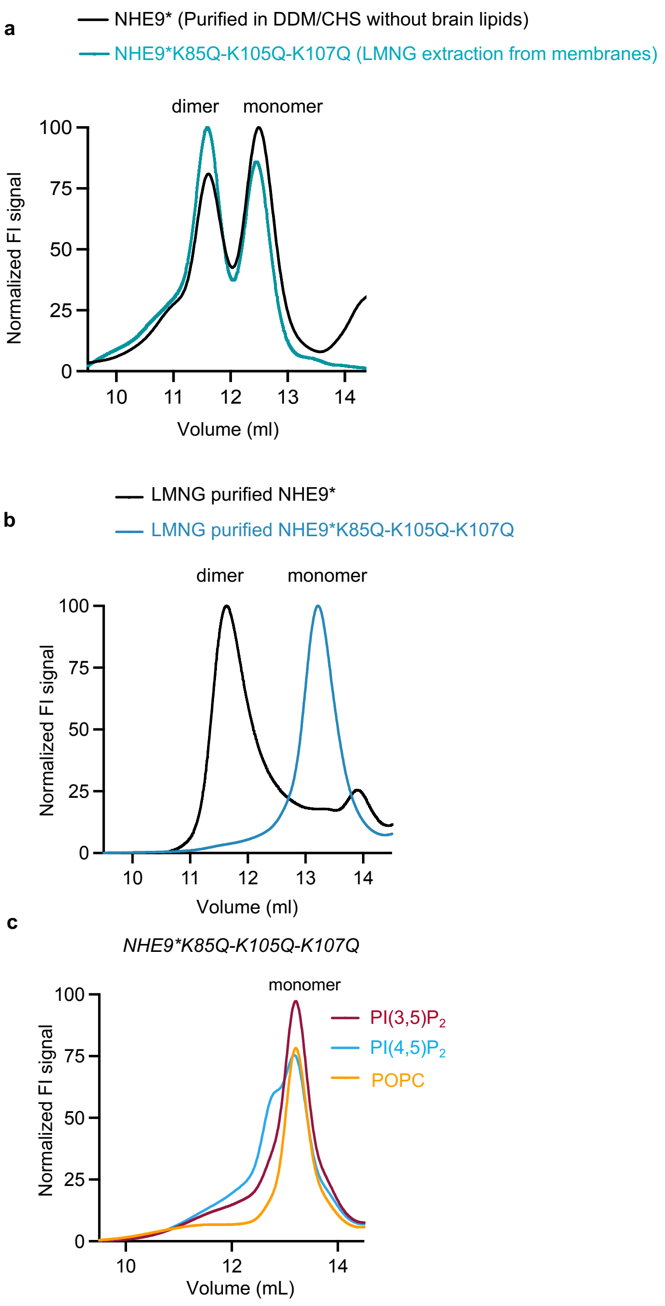

Supplementary Fig. 8. NHE9* and varaints analysed by FSEC. a. Representative normalized FSEC traces for purified NHE9*-GFP fusion (black) purified in DDM/CHS without addition of brain lipids and NHE9*(K85Q, K105Q, K107Q)(blue) following membrane extraction in LMNG. **b.** Representative normalized FSEC traces for LMNG purified NHE9*-GFP fusion (black) and NHE9*(K85Q, K105Q, K107Q)-GFP fusion (blue). **c**. Representative normalized FSEC traces for purified NHE9*(K85Q, K105Q, K107Q)-GFP fusion after heating at 50ºC for 10 mins in the presence of DDM-solublised lipids as labelled.

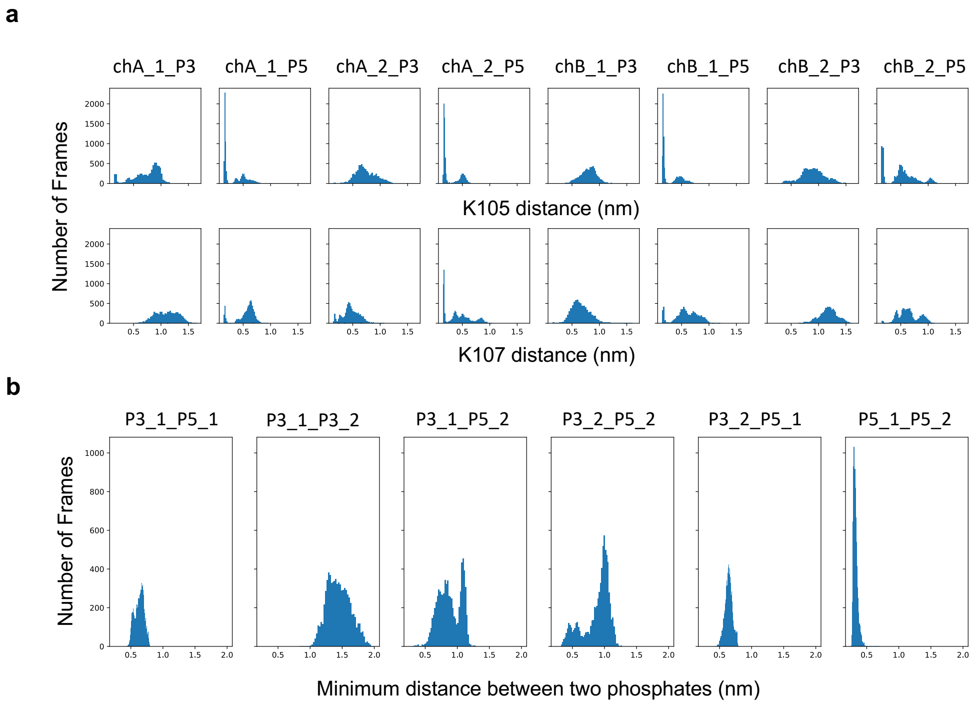

Supplementary Fig. 9. Distribution of distances between TM2-TM3 β-hairpin lysines and phosphate groups of the PI(3,5)P2 lipids. **a.** The distribution of minimum distances between K105 or K107 on the first subunit (chA) or the second subunit (chB) of NHE9 and with 3'phosphate (P3) or 5'phosphate (P5) on the first PIP(3,5)P2 lipid (1) or the second PIP(3,5)P2 lipid (2). For example, the chA_1_P5 denotes the distribution of the minimum distance between K105 on the first subunit of NHE9 interaction with the 5'phosphate on the first PIP(3,5)P2 lipid. The triplicates of the simulation data are combined into one histogram. **b**. The distribution of distances between 3'phosphate (P3) or 5'phosphate (P5) on the first PIP(3,5)P2 lipid (1) or the second PIP(3,5)P2 lipid (2). For example, the P3_1_P5_2 denotes the distribution of the minimum distance between 3'phosphate on the first PIP(3,5)P2 with the 5'phosphate on the second PIP(3,5)P2 lipid. The triplicates of the simulation data are combined into one histogram.

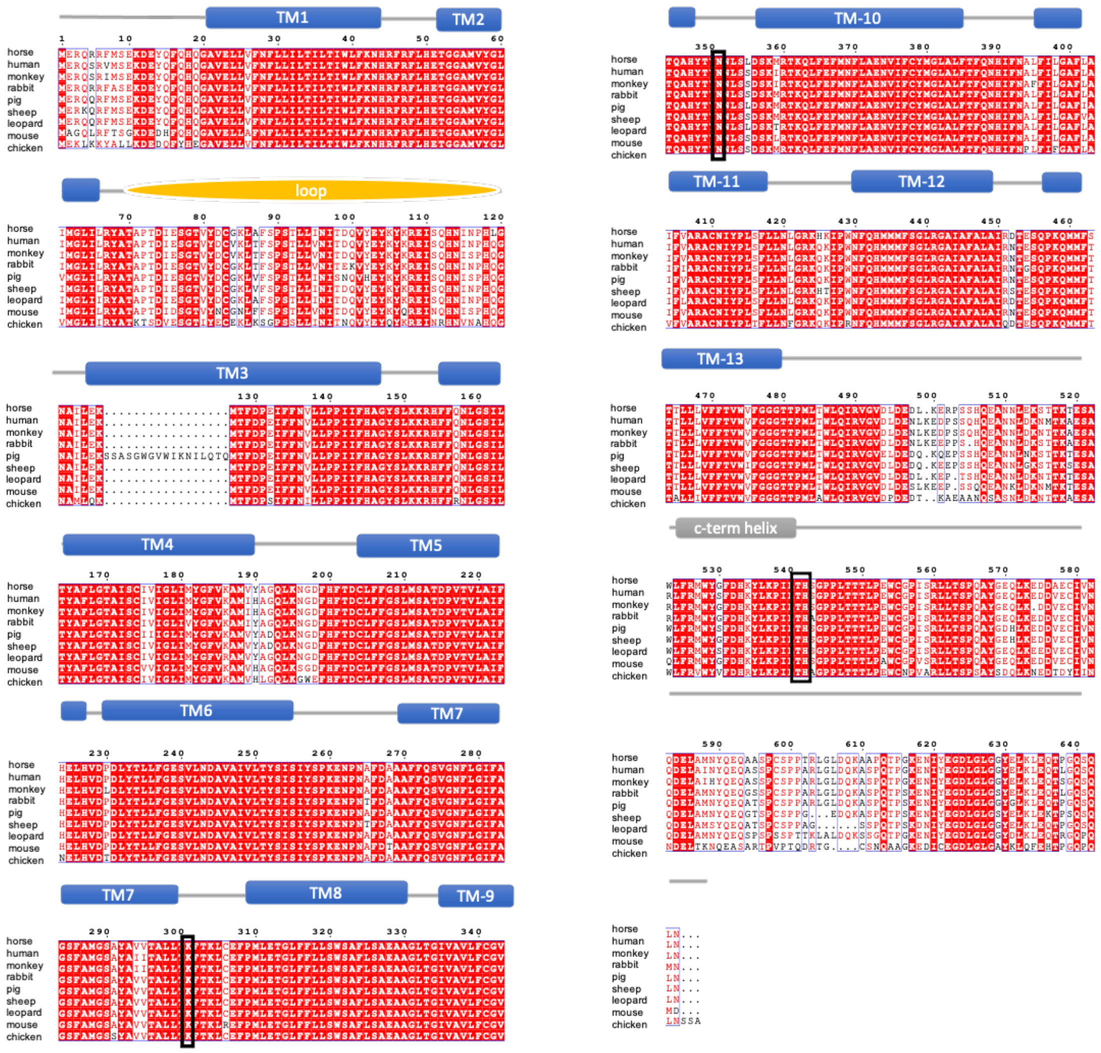

Supplementary Fig.10. Multiple sequence alignment for NHE9 among vertebrates. The following are the accession ID's for *E.caballus* (F7B113), human (Q8IVB4), monkey (H9FT94), rabbit (G1SY59), pig (A0A287BEB2), sheep (W5P6Y8), leopard (A0A6P4V361), mouse (Q8BZ00), chicken (A0A8V0YVM6),. Residues with over 90% sequence identity are indicated by red background. Positions which are involved in gating (K301) and c-terminus regulation (T541, H542) are highlighted in black box. Residues which form TMs (blue), Loop (yellow) and C-term helix (grey) are indicated.

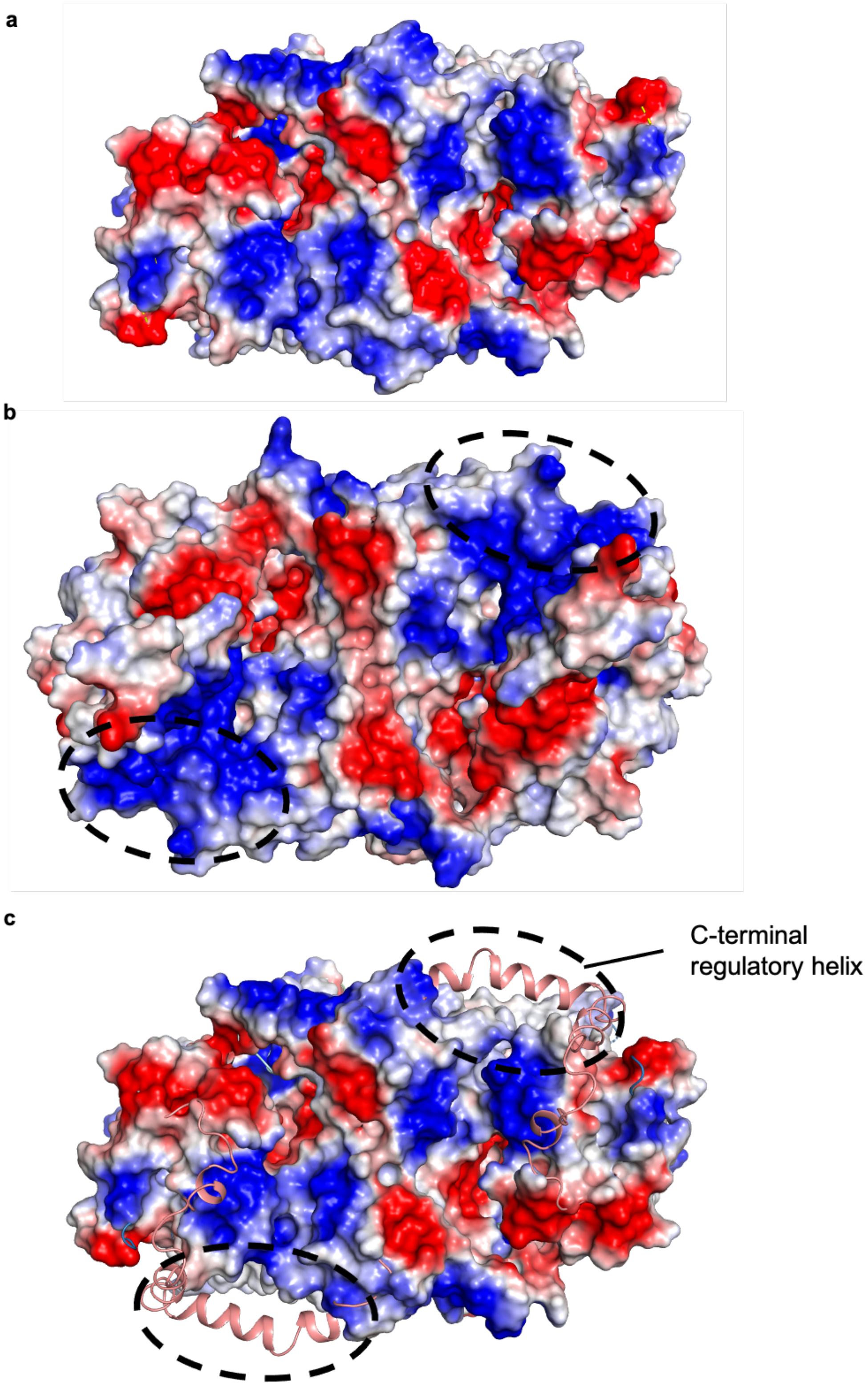

Supplementary Fig. 11. Visualizing the difference upon inclusion of the C-terminal regulatory domain on overall structure of NHE9 viewed from the cytosloic side. a Previous published structure of NHE9* (PDB id: 6Z3Z) where the CTD could not be modelled shown in as an electrostatic surface potential. **b.** Improved NHE9* CC

structure with CTD density (black dashed circle) (PDB id; 8PVR). **c.** Improved NHE9* CC structure shown as in A, with the additional CTD helix shown as cartoon (salmon), but without its electrostatic surface (black dashed circle).

	**NHE9*loop (EMDB-18002)(PDB 8PBX)**	**NHE9*CC (EMDB-17971)(PDB 8PVR)**	***Ec*NhaA-mut2 (EMBD-17841)(PDB 8PS0)**
Data collection and processing			
**Magnification**	165,000	130,000	130,000
**Voltage (kV)**	300	300	300
**Electron exposure (e–/Å^2)**	57	68.5	68.1
**Defocus range (µm)**	0.9-2.3	0.4-2.0	0.6-2.0
**Pixel size (Å)**	0.82	0.66	0.6645
**Symmetry imposed**	C2	C2 (and C1)	C2
**Initial particle images (no.)**	2,360,025	4,421,363	4,401,429
**Final particle images (no.)**	103,815	78,370	78,917
**Map resolution (Å)**<br>**FSC threshold**	3.6<br>0.143	3.15<br>0.143	3.37<br>0.143
**Map resolution range (Å)**	3.2-4.1	2.5-3.5	2.4-4.0
Refinement			
**Initial model used (PDB code)**	NHE9*CC and NHE9 Alphafold2 dimer	6Z3Z and NHE9 Alphafold2 dimer	7S24

**Model resolution (Å)**	4.0	3.5	3.8
**FSC threshold**	0.5	0.5	0.5
**Model resolution range (Å)**	3.2-4.0	2.5-3.5	2.5-4.0
**Map sharpening *B* factor ($Å^2$)**	-	-	-
**Model composition**			
**Non-hydrogen atoms**	8188	8506	5739
**Protein residues**	1018	1058	744
**Ligands**	-	$PI(3,5)P_2$	CDL:3
***B* factors ($Å^2$)**			
**Protein**	103.73	165.98	92.07
**Ligand**		221.06	161.24
**R.m.s. deviations**			
**Bond lengths (Å)**	0.002	0.002	0.002
**Bond angles (°)**	0.441	0.508	0.638
**Validation**			
**MolProbity score**	1.58	1.80	1.73
**Clashscore**	6.30	7.95	6.59
**Poor rotamers (%)**	1.11	0.00	0.86
**Ramachandran plot**			
**Favored (%)**	96.44	94.65	94.58
**Allowed (%)**	3.56	5.35	5.42
**Disallowed (%)**	0	0	0

Supplementary Table 1. Data collection, processing and refinement statistics of NHE9*loop, NHE9*CC and *Ec*NhaA-mut2 cryo EM structures.
